# High Electron Mobility in Ge Films Grown on Si (001) by an 8-Inch Molecular Beam Epitaxy System

**DOI:** 10.3390/nano16070424

**Published:** 2026-03-31

**Authors:** Gancheng Ye, Jieyin Zhang, Yilin Chen, Ming Ming, Liangxin Liao, Xin Geng, Xinding Zhang, Jianjun Zhang

**Affiliations:** 1 School of Physics, South China Normal University, Guangzhou 510631, China; 2 Songshan Lake Materials Laboratory, Dongguan 523808, China; 3 Institute of Physics, Chinese Academy of Sciences, Beijing 100190, China; 4 School of Integrated Circuits, Sun Yat-Sen University, Shenzhen 518107, China

**Keywords:** Ge film, molecular beam epitaxy, hall effect measurements

## Abstract

Silicon-based germanium films are promising for the fabrication of low-power, high-performance electronic and optoelectronic devices. In this work, we report an effective approach for directly growing Ge films with ultrahigh carrier mobility on Si (001) substrates using molecular beam epitaxy (MBE). Strain relaxation of the germanium films is realized through the formation of partial dislocations and 90° misfit dislocations at the Ge/Si interface. The Ge film exhibits a smooth surface with a root-mean-square roughness of 0.187 nm and a low threading dislocation density of only 1.2 × 10^7^ cm^−2^. Hall effect measurements reveal a high room-temperature mobility of up to 1916 cm^2^V^−1^s^−1^ along with a carrier concentration of 1.425 × 10^16^ cm^−3^. These findings demonstrate that MBE-grown Ge films, possessing exceptionally high carrier mobility, hold great promise for integration into advanced electronic and optoelectronic devices.

## 1. Introduction

Germanium exhibits higher carrier mobility than silicon [1] and is compatible with traditional silicon processing technologies, making it a promising candidate for low-power electronic devices and optoelectronic devices. In the field of electronic devices, silicon-based germanium has been widely investigated for Ge-based MOSFETs for low-power logic [2,3] and Ge-based FinFET devices [4]. In optoelectronics, high-performing germanium-silicon single-photon detectors [5] based on silicon-based germanium have emerged as promising candidates for short-wave infrared single-photon detection. Additionally, Ge-on-Si lasers [6] offer a viable route toward high-volume electronic–photonic integration, while Ge-on-Si p-i-n photodetectors [7] enable high-speed operation and high sensitivity for optical data communication. These developments underscore the importance of silicon-based germanium in advanced optoelectronic applications.

However, the 4.2% lattice mismatch between Ge and Si poses challenges in achieving high crystalline quality and smooth surfaces in epitaxial Ge films grown on silicon substrates. Various approaches—such as compositionally graded buffers [8], patterned substrates [9], and two-step growth methods [10]—have been developed to reduce threading dislocation density (TDD). Even the chemical mechanical polishing (CMP) process was applied to reduce the surface roughness [11,12].

In this study, we demonstrate a repeated low-temperature epitaxy and high-temperature annealing process using an 8-inch MBE system, achieving fully relaxed Ge film with a root-mean-square (RMS) roughness of 0.187 nm and a threading dislocation density of 1.2 × 10^7^ cm^−2^. Hall effect measurements of the films yield a room-temperature electron mobility of 1916 cm^2^V^−1^s^−1^ and a carrier concentration of 1.425 × 10^16^ cm^−3^, highlighting the potential of MBE-grown Ge films for high-performance device applications.

## 2. Experiment

Undoped Ge films were grown on Si (001) substrates by MBE with a background vacuum level of 10^−11^ mbar. Ge was evaporated by e-gun and high-purity Ge source materials with a purity of 8 N (99.999999%) produced by Vital Optics Technology Co., Ltd. of China (Chuzhou, China). Prior to loading, an 8-inch Si wafer with resistivity of 10,000 ohm·cm were dipped in a diluted HF solution (~4 vol%) for 1 min to remove native oxide and passivate the surface with hydrogen. After rinsing with deionized water and drying with N_2_, the substrate was loaded to the MBE chamber and degassed at 400 °C and 550 °C. A 200 nm Ge layer was first deposited at 300 °C, annealed in the temperature range of 700–730 °C for 10 min, and then followed by eight cycles of 100 nm Ge deposition at 300 °C and 700–730 °C annealing for 10 min, resulting in a total thickness of ~1 µm, and the growth temperature of the final 50 nm Ge layer was raised to 400 °C. Surface morphology was analyzed by atomic force microscopy (AFM, Bruker, Santa Barbara, CA, USA). Dislocation characterization of the sample was performed using electron channeling contrast imaging (ECCI, ZEISS, Oberkochen, Germany) and X-ray diffraction (XRD, Malvern Panalytica, Almelo, The Netherlands) Omega scans. The stress of Ge film was measured using X-ray diffraction reciprocal space mapping (XRD-RSM). Cross-sectional structure and interface were examined by transmission electron microscopy (TEM, JEOL, Tokyo, Japan).

Hall-bar devices were fabricated from 1 µm thick Ge film grown on Si substrate. First, Hall-bar mesas were defined by direct laser writing (DLW) and reactive ion etching (RIE). The longitudinal length (L) and transverse width (W) of the Hall bars were set at 370 µm and 100 µm, respectively. In the contact regions, 70 nm of Pt was deposited by electron-beam evaporation, followed by annealing at 350 °C for 2 h to form ohmic contacts. Temperature-dependent Hall measurements were carried out in a six-probe configuration using a cryogenic system equipped with a 9 Tesla superconducting magnet. A 1 V AC voltage at a frequency of 13.3333 Hz was applied between the source and drain contacts, with a 10 MΩ resistor connected in series in the circuit.

## 3. Results and Discussion

**Figure 1:**
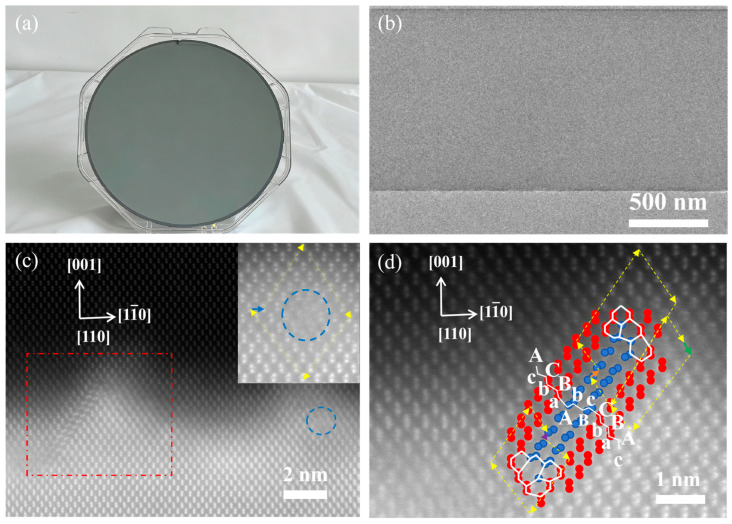
(**a**) Photograph of an 8-inch silicon-based germanium film sample. (**b**) Cross-sectional TEM image of the Ge film grown on Si (001). (**c**) HAADF-STEM image of the Ge/Si (001) interface area, the stacking fault and the 90°dislocation are enclosed by the red dashed rectangle and the blue circle, where the Burgers circuit and Burgers vector are indicated by yellow and blue arrows. (**d**) 30° (the purple arrow), 60° (the green arrow) and 90°dislocations (the orange arrow) and stacking fault (the blue atoms) at the interface. The [110] crystallographic direction is perpendicular to the page, pointing out of the plane.

Figure 1a shows an undoped Ge film sample grown on an 8-inch Si (001) substrate using MBE. Figure 1b shows the cross-sectional transmission electron microscopy image of the Ge film, in which no threading dislocations are observed. Figure 1c,d show HAADF–STEM images of the Ge/Si heterointerface. Figure 1c presents a 90° misfit dislocation, where the Burgers circuit and Burgers vector are indicated by yellow and blue arrows, respectively. The 90° misfit dislocation has a Burgers vector $\vec{b}=\frac{1}{2}[1\bar{1}0]$ and is formed by the interaction of two 60° misfit dislocations with Burgers vectors in different glide planes $\frac{1}{2}[101]+\frac{1}{2}[0\bar{1}\bar{1}]\to \frac{1}{2}[1\bar{1}0]$ [13]. The majority of strain relaxation is realized through the formation of 90° misfit dislocations at the Ge/Si interface, which are mainly distributed on the 001 plane at the Ge/Si interface, resulting in high crystal quality with low threading dislocations density. As shown in Figure 1d, the yellow arrow marks the Burgers circuit, the green arrow indicates the perfect 60° dislocation with Burgers vector $\vec{b}=\frac{1}{2}\left[0\bar{1}\bar{1}\right].$ Figure 1d also shows the enlarged view of the stacking-fault region delineated by the red dashed box in Figure 1c. In the SF region (Figure 1d), the stacking sequence changes from AaBbCcAaBbCc to AaBbCcBbAaBbCc, where “Bb” denotes an inserted plane. Accordingly, the presence of this additional plane identifies the defect as an extrinsic stacking fault. The orange arrow denotes the 90° partial dislocation with Burgers vector $\vec{b}=\frac{1}{6}[1\bar{1}2]$, and the purple arrow denotes the 30° partial dislocation with Burgers vector $\vec{b}=\frac{1}{6}[211]$. The core of the 90° partial dislocation is composed of six-membered atomic rings, whereas the core of the 30° partial dislocation comprises a five-membered ring, a six-membered rings, and a seven-membered ring [14]. This configuration is in agreement with the model proposed by Lu and Smith for a partial dislocation bounded by an extrinsic stacking fault [15]. Under compressive strain, a perfect 60° misfit dislocation dissociates into two Shockley partial dislocations, which is given by $\frac{1}{2}\left[101\right]\to \frac{1}{6}\left[1\bar{1}2\right]+\frac{1}{2}[211]$. The 30° partial dislocation and the 90° partial dislocation both release part of the internal stress within the film [14,16].

**Figure 2:**
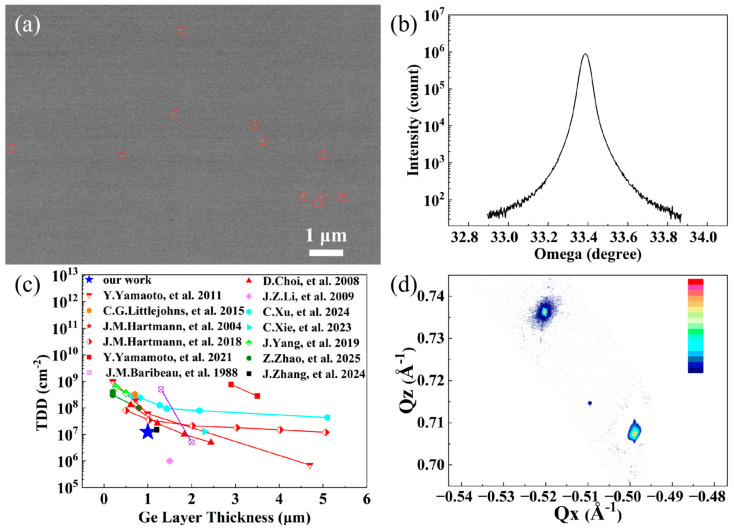
(**a**) ECCI measurements of the Ge film at an accelerating voltage of 5 kV, the threading dislocations are marked by the red circles. (**b**) (004) Omega rocking curve of the Ge film. (**c**) Threading dislocation density as a function of Ge film thickness. Data are acquired from the literatures [9,10,11,18,20,21,22,23,24,25,26,27,28]. (**d**) XRD-RSM of the Ge film grown on Si (001).

Figure 2a shows the ECCI characterization of the surface of the Ge film acquired at an accelerating voltage of 5 kV. For the Ge film, the average effective penetration depth of backscattered electrons at a low accelerating voltage of 5 kV is approximately 50 nm [17]. The measured threading dislocation density is approximately 1.2 × 10^7^ cm^−2^. Figure 2b shows the (004) Omega rocking curve of the Ge film measured by XRD. The rocking curve full width at half maximum (FWHM) is 158 arcseconds. Using the relation $\mathrm{TDD}\;=\;1632\;\times \;\beta^{2}$, the threading dislocation density is calculated to be 4 × 10^7^ cm^−2^ [18]. The threading dislocation density obtained by ECCI corresponds to an effective penetration depth of approximately 50 nm from the surface, whereas XRD measurements reflect the dislocation density of the entire Ge film, especially including the interface area, which means that the rocking curve contains information about interface misfit dislocations and their correlations [19]. As a result, the TDD determined by ECCI is lower than that obtained from XRD. Figure 2c compares the threading dislocation density of the Ge film in this work with those of Ge films reported in previous studies [9,10,11,18,20,21,22,23,24,25,26,27,28]. In this work, the Ge film underwent prolonged high-temperature annealing during growth, which effectively reduced the dislocation density and resulted in superior crystalline quality compared with other reported studies. Figure 2d presents the reciprocal space map (RSM) of the (−2–24) reflection measured by XRD. The film relaxation is calculated to be R = 102.4%. The tensile strain on the Ge film results from the cooling process after annealing, where germanium, with a room-temperature thermal expansion coefficient of 5.8 × 10^−6^ K^−1^, undergoes a larger contraction than the silicon substrate with 2.59 × 10^−6^ K^−1^ [29,30].

**Figure 3:**
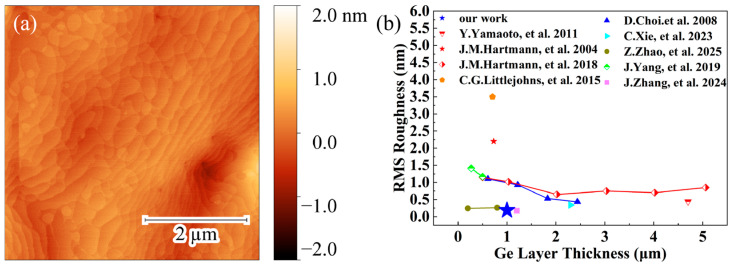
(**a**) AFM surface image of the MBE-grown Ge film on Si (001). (**b**) RMS roughness as a function of Ge film thickness. Data are acquired from the literatures [10,11,18,21,22,23,25,26,28].

Figure 3a shows the AFM characterization of the surface of the Ge film on a Si (001) substrate. Clear atomic steps are observed on the surface. The RMS roughness of the sample surface is only 0.187 nm, indicating a smooth Ge film surface. Figure 3b compares the surface RMS roughness of the Ge film on Si in this work with those reported by previous studies [10,11,18,21,22,23,25,26,28]. The Ge film in our work exhibits a lower root-mean-square surface roughness due to the low-temperature growth of Ge to reduce Ge adatom surface diffusion.

**Figure 4:**
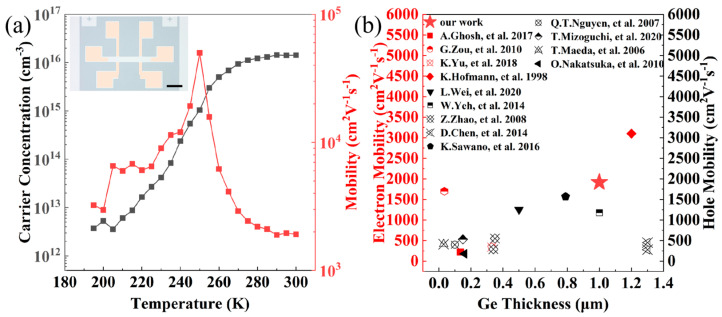
(**a**) Temperature-dependent variation in the carrier concentration and carrier mobility of the sample along the <110> crystallographic direction. The inset shows the optical image of the Hall-bar device, where the black scale bar corresponds to 250 μm. (**b**) Carrier mobility in Ge film on Si as a function of Ge thickness from data acquired in this work and other research groups [31,32,33,35,36,37,38,39,40,41,42,43,44]. Electron mobility is highlighted in red, while hole mobility is highlighted in black.

Hall effect measurements were performed on the Ge film using a Hall-bar configuration. The inset in Figure 4a shows an optical micrograph of the fabricated Hall-bar device, with the channel oriented along the <110> direction. Figure 4a presents the temperature-dependent carrier concentration (red squares) and carrier mobility (black squares) of the Ge film. As the temperature decreases, the carrier mobility increases, reaching a maximum, and then decreases, while the carrier concentration decreases monotonically. The sample exhibits n-type conduction behavior due to the unintentional doping during the growth. At 250 K, the electron mobility reaches a maximum value of 49,990 cm^2^ V^−1^ s^−1^, corresponding to a carrier concentration of 1.041 × 10^15^ cm^−3^. At room temperature, the electron mobility is 1916 cm^2^ V^−1^ s^−1^, with a carrier concentration of 1.424 × 10^16^ cm^−3^.

The decrease in carrier concentration with decreasing temperature is attributed to carrier freeze-out, where thermally activated carriers are progressively trapped by impurity and defect states [31]. In the low-temperature regime (195–250 K), both the carrier mobility and carrier concentration increase with increasing temperature. The reasons behind the deviation of the power-law exponent relating mobility and temperature from the values (3/2 and 1) expected for impurity scattering and dislocation scattering remain unclear. Further experimental investigations are needed to clarify this issue. In the high-temperature regime (250–300 K), the mobility begins to decrease as the temperature rises, implying that acoustic- and optical-phonon scattering become significant as the temperature approaches room temperature [32,33,34]. Figure 4b compares the room-temperature carrier mobility of the Ge film on Si obtained in this work with values reported in previous studies by other research groups [31,32,33,35,36,37,38,39,40,41,42,43,44]. The Ge film in this study exhibits significantly higher electron mobility, which can be attributed to its superior crystalline quality and the consequent reduction in dislocation and impurity scattering resulting from the application of high-purity Ge source and the control of ultra-high vacuum.

## 4. Conclusions

In summary, we obtained a high-quality Ge film on silicon substrates through multiple low-temperature epitaxy and high-temperature annealing using an 8-inch molecular beam epitaxy system. AFM characterization indicates a smooth surface with a root-mean-square roughness of 0.187 nm, while ECCI measurements reveal a threading dislocation density of 1.2 × 10^7^ cm^−2^. Hall effect measurements of the films yield a room-temperature electron mobility of 1916 cm^2^V^−1^s^−1^ and a carrier concentration of 1.425 × 10^16^ cm^−3^. Our work indicates that Ge films grown via MBE exhibit high carrier mobility and superior crystalline quality, making them highly promising for applications in both integrated circuits and silicon-based optoelectronic integration.

## Data Availability

The original contributions presented in this study are included in the article. Further inquiries can be directed to the corresponding authors.
